# Perspectives of service providers on aftercare service provision for persons with substance use disorders at a Rural District in South Africa

**DOI:** 10.1186/s13011-022-00471-5

**Published:** 2022-08-12

**Authors:** December Mandlenkosi Mpanza, Pragashnie Govender, Anna Voce

**Affiliations:** 1 Discipline of Occupational Therapy, School of Health Sciences, University of KwaZulu Natal, Private Bag x54001, Durban, 4000 South Africa; 2 Discipline of Public Health Medicine, School of Nursing and Public Health, University of KwaZulu Natal, Private Bag x54001, Durban, 4000 South Africa

**Keywords:** Aftercare, Service provision, Persons with substance use disorders (PWSUD)

## Abstract

**Background:**

Provision of aftercare services for persons with substance use disorders (PWSUD) within a rural context is typically met with various intersecting challenges, including unclear policy implications and lack of resources. In the South African context, service providers are expected to provide aftercare services that should successfully reintegrate persons with PWSUD into society, the workforce, family and community life as mandated by Act No. 70 of 2008, despite population diversity. Little has been established on the provision of aftercare services in South Africa and specifically within a rural context. This article explores service providers’ perspectives in aftercare service provision for PWSUD in a rural district.

**Methods:**

A qualitative exploratory study design was conducted in a rural district in South Africa using semi-structured interviews and focus group discussions with forty-six service providers from governmental and non-governmental institutions, ranging from implementation to policy level of service provision. Data were analyzed thematically using a deductive approach. Codes were predetermined from the questions and the aims and objectives of the study used Beer’s Viable Systems Model as a theoretical framework. NVivo Pro 12 qualitative data analysis software guided the organization and further analysis of the data.

**Results:**

Four themes emanated from the data sets. Theme 1 on reflections of the interactional state of aftercare services and program content identified the successes and inadequacies of aftercare interventions including relevant recommendations for aftercare services. Themes 2, 3, and 4 demonstrate reflections of service provision from implementation to policy level, namely, identifying existing barriers to aftercare service provision, situating systemic enablers to aftercare service provision, and associated aftercare system recommendations.

**Conclusions:**

The intersecting systemic complexities of providing aftercare services in a rural context in South Africa was evident. There existed minimal enablers for service provision in this rural district. Service providers are confronted with numerous systemic barriers at all levels of service provision. To strengthen the aftercare system, policies with enforcement of aftercare services are required. Moreover, a model of aftercare that is integrated into the existing services, family centered, sensitive to the rural context and one that encourages the collaboration of stakeholders could also strengthen and sustain the aftercare system and service provision.

## Background

Provision of aftercare services for persons with substance use disorders (PWSUD) within a rural context is typically met with various intersecting challenges, including unclear policy implications and scarcity of resources [1–3]. These challenges contribute to a decrease of recovery capital, of which aftercare is critical in increasing recovery capital for PWSUD [4]. However, a strategically planned aftercare program, also known as recovery management, is pertinent for improved and sustained treatment outcomes, particularly relapse prevention [5–7]. Globally, 60% of PWSUD relapse due to limited and inadequate treatment services and poor aftercare services [6] and one out of seven PWSUD have access to treatment [8]. In low-and middle-income countries (LMICs), especially in Sub-Saharan Africa, including South Africa (SA), one out of eighteen persons have access to treatment [6]. Notwithstanding this, where treatment is available, it is often not evidence-based and ineffective [6]. Consequentially, post-discharge relapse and re-admission rates remain consistently high, with an estimated 40% of PWSUD with sustained recovery [6]. Aftercare service provision is limited globally [6, 8, 9] due to several prevailing barriers. Predominantly, there is a lack of scientific evidence on aftercare and long-term recovery management, as the efficacy of interventions has not been assessed in most countries [6]. The World Health Organization (WHO) and the United Nations Office on Drugs and Crime (UNODC) have in fact, promulgated treatment guidelines, recently published in 2020, which included aftercare. Still, these guidelines have only been tested scientifically in ten countries thus far [7]. However, contextual barriers are confronting each country in aftercare service provision. In SA, there exists a lack of distinctive policy directives on aftercare services which may be attributed to a lack of empirical evidence within these policies [3]. The overall guiding policy, the National Drug Master Plan 2019–2024, only provides a superficial guide to aftercare services.

Aftercare service provision is a sub-system of the SUD treatment service provision system in SA [8], an overall treatment intervention system for people with SUD. The SUD treatment service provision is characterized by several complexities compounded by the two separate systems of service provision, namely, public and private healthcare systems [3]. In the public sector, the tax-funded healthcare system services are provided to the majority of the population (about 86%) at no cost, particularly for those who are indigent or on an income-based scale [10]. Services in the private sector, which serve about 16% of the population, are expensive, therefore the majority of PWSUD utilize their medical aids/personal medical insurance to pay for services [3, 10]. The public sector services are generally limited and inaccessible to the majority of South Africans, in particular the rural population [2]. For instance, the KwaZulu-Natal (KZN) Province, the second-largest province in SA where this study was conducted, has only two public sector in-patient treatment centers located in urban areas.

In contrast, there has been an increase in the establishment of private sector treatment services (both licensed and unlicensed) in SA, with at least one facility in each city [2]. In addition, policy reforms such as the White Paper for the Reconstruction and Development Program (1994) and the White Paper for Social Welfare (1997), were intended to hasten transformation from a social welfare state to a developmental state and a developmental approach to the provision of social welfare services [3], which has a preferential focus on those most vulnerable and disadvantaged [11]. The developmental approach is person- centered, promoting self-reliance and the capacity for growth and development through using own strengths, knowledge and maximizing human potential [11]. Furthermore, services are integrated, family-centered and community-based [11]. Also, the National Drug Master Plan (NDMP) 2019–2024, an overall policy on SUD, promotes community-based service provision to all people within their areas of residence [12]. Paradoxically, disadvantaged communities continue to experience high levels of unmet treatment needs, which is a burden to the health and welfare system of South Africa [13]. Largely, the origins of these unmet treatment needs can be traced to the general inequitable spread and limited availability of substance abuse treatment services in SA [2]. Coherently, the SA population remains the world’s most unequal nation [14], with 40% of the SA population residing in rural areas [15]. Therefore, there may be additional complexities to the SUD treatment service provision system in SA that would require further exploration.

Little has been established on aftercare services provision in SA and specifically in rural contexts. Most SA studies [1, 2, 13, 16] have examined the treatment service provision for SUD with limited comments on aftercare. The few studies [17–20], that focused on aftercare were urban-based. Therefore, the extent to which the findings of these studies [17–20] apply to the rural context remains unknown. An extensive literature search revealed that no South African studies had examined aftercare service provision in a rural context; it is, therefore, pertinent to extrapolate this study to the rural context of SA.

### Problem statement

The province of KZN, which includes numerous rural areas [21], experiences various barriers in service provision. There are lengthy waiting lists for admission to the only two available public in-patient treatment centers (ITCs) [22], both located in urban areas. Therefore, the population in rural districts is required to travel to access ITCs before being discharged back into their community for aftercare services [23]. These ITCs are mostly unavailable or inadequate [16, 22, 23] and is a scenario applicable to the rural district in which this study was located. Aftercare services in this district are limited, inadequate and fragmented among stakeholders, with no monitoring and evaluation [1].

Consequently, some PWSUD are often lost in the system of care and re-emerge following a relapse [1]. A recent study by Mpanza et al., [24] on the same district revealed numerous inadequacies reported by PWSUD. These include the majority of PWSUD not accessing aftercare, and those who received aftercare experienced services that were terminated abruptly. The reasons for such inadequacies of aftercare and how service providers navigate such barriers are not well documented. Ultimately, service providers are expected to provide an aftercare service that should successfully reintegrate PWSUD into their society, the workforce, family and community life as mandated by Act No. 70 of 2008 [25]. In earlier work by Mpanza et al., an analysis of the policy context in SA was done [3], followed by an exploration of the aftercare service provision from the perspectives of PWSUD. In this current study, the service providers’ perspectives in aftercare service provision for PWSUD were explored in this rural district. This exploration is underpinned by the justification that a comprehensive investigation into the state of aftercare services provision may assist in informing policy developments. It may also assist in improving service delivery strategies, thereby ultimately improving the aftercare service provision within the health system.

## Methods

### Theoretical framework

Aftercare services for PWSUD is an intrinsic sub-system in the component of the service provision system. However, the coordination of aftercare provision is perceived as a system endemic to the SUD service provision, comprising multiple components and interactions with the associated complexity of human services systems. With this understanding, systems thinking, also known as a systems approach, was utilized as the theoretical framework underpinning this study. Systems thinking focuses on the whole and the interactions/relationships of its parts/sub-systems [26–28] instead of understanding singular components that ignore the holistic interaction between systems. Moreover, systems thinking emphasizes cross-sector collaboration and community partnerships [29], and the NDMP of 2019–2024 supports the systems approach in preventing relapse during aftercare [12]. Hence, the systems approach was adopted in this study.

Within systems thinking, the study was additionally framed using the Beer’s Viable Systems Model (VSM) which facilitated in-depth exploration of the interactions and roles fulfilled by each component for the effective functioning of the system [27, 28]. The Beer’s VSM has five components, namely, implementation (institution where PWSUD are serviced), coordination (district and institution), control (district level), development/intelligence (provincial) and policy (provincial and national) within a given environment. In applying the VSM model, an exploration of how these five components relate to the aftercare service provision system is provided.

The study was situated within a social constructivist paradigm. It sought to understand the world through the perspectives and constructed meanings (often complex and varied) of the participants’ experiences of their lived realities [30]. This article is a component of a larger study that seeks to inform a model for aftercare service provision, and preceded by a policy analysis within the SA context [3] and perspectives of PWSUD on aftercare needs [24].

### Study design

A qualitative exploratory study design was adopted to gain a comprehensive understanding of the aftercare service provision from the perspectives of services providers for PWSUD. Such a design is appropriate where limited knowledge exists and facilitates a more in-depth exploration of the context to discover social meaning and its impact on individuals [31].

### Location of the study

The study was conducted at a rural district, one of eleven districts in KZN with a total population of 11,3 million people, equating to approximately 19,2% of the South African population [15]. KZN has more than 55% of the population domiciled in rural areas [21], with 25% of disadvantaged South Africans (57% below the poverty line) residing in the province [15]. The province has the triple burden of high poverty levels, HIV and Tuberculosis (TB) [32], complicating service provision. This particular rural district has between 82 and 95% of households who live below the poverty line, with 70% of this population living on less than 800 South African Rands (ZAR) (54$) per month [33]. No ITCs are located in this district; resources are limited with poor infrastructure (predominantly gravel roads) similar to most rural areas in SA. Hence PWSUD are referred to the two urban public ITCs. Located within this district, are public hospitals, one NGO providing outpatient mental healthcare treatment services (including services for SUD) among other programs, and an additional NGO providing outpatient services for SUD and referrals for in-patient intervention to public and private ITCs (to those who can afford the service). The district also has several social service centers providing social welfare services, including outpatient services. There are several shebeens providing illicit alcohol and other home-brewed substances and cannabis (mainly cultivated at home) commonly used in this district [1]. Consistently, the treatment center admissions in KZN indicate that cannabis, the most common substance of abuse, accounted for 37% of admissions, followed by heroin (including N*yaope* / W*hoonga*) at 27% and alcohol at 14% [34].

### Sampling strategy

Non-probability maximum variation purposive sampling was used to recruit and select participants (*n* = 45) who represented all five levels of the Beer’s VSM [26] and sectors within the SUD system of service provision (Table 1). The selection criteria included service providers who were employed for at least 1 year in SUD-related service provision at any level of the service provision system. The final sample size was achieved following data saturation, where the iterative collection and analysis of data presented no new information.

**Table 1:** Categories of Stakeholder groups represented within levels of function in Beer’s VS

LEVEL OF FUNCTION	ACTOR’S DESIGNATION	DISCIPLINE	ITC (DSD)	DSD	DoH	NGO1	NGO2	TOTAL
Policy/Intelligence/ Development) *n* = (10)	Executive Director	Social Worker				**1**		**1**
	Director	Project Management					**1**	**1**
	Substance Abuse Services Director	Social Worker		**1**				**1**
	Deputy District Manager/Programs Manager	Social Worker		**3**				**3**
	Substance Abuse District Coordinator	Social Worker		**1**				**1**
	Clinical and Programs Manager	Nursing			**1**			**1**
	Facility Manager	Social Worker	**2**					**2**
Control/Monitoring and Evaluation) (*n* = 8)	Substance Abuse Facility Coordinator	Social worker		**3**				**3**
	Mental Health and Rehabilitation Coordinator	Occupational Therapist			**1**			**1**
	Social Work Supervisor	Social Worker	**1**					**1**
	Head of Social Services	Social Worker			**1**			**1**
	Medical Manager	Medical Officer			**1**			**1**
	Head of Occupational Therapy Department	Occupational Therapist			**1**			**1**
Implementation/Service provision level *n* = 28	Counseling Psychology Service Provider	Counseling Psychologist					**1**	**1**
	Social Auxiliary Service Provider	Social Auxiliary		**1**				**1**
	Occupational Therapy Service Provider	Occupational Therapist	**1**		**2**			**3**
	Occupational Therapy Technician Service Provider	Occupational Therapy Technician			**1**		**1**	**2**
	Social Work Service Provider	Social Worker	**2**	**1**	**5**	**3**	**2**	**13**
	Mental Healthcare Service Provider	Nursing			**4**		**1**	**5**
	Counselling Service Provider	Lay counsellor					**1**	**1**
	Nursing Services	Nursing	**2**					**2**
**Grand Total**			**8**	**10**	**17**	**4**	**7**	**46**

*ITC* Inpatient Treatment Centre, *DSD* Department of Social Development, *DoH* Department of Health, *NGO1 and NGO2* Non-Governmental Organizations

### Data generation

**Fig. 1:**
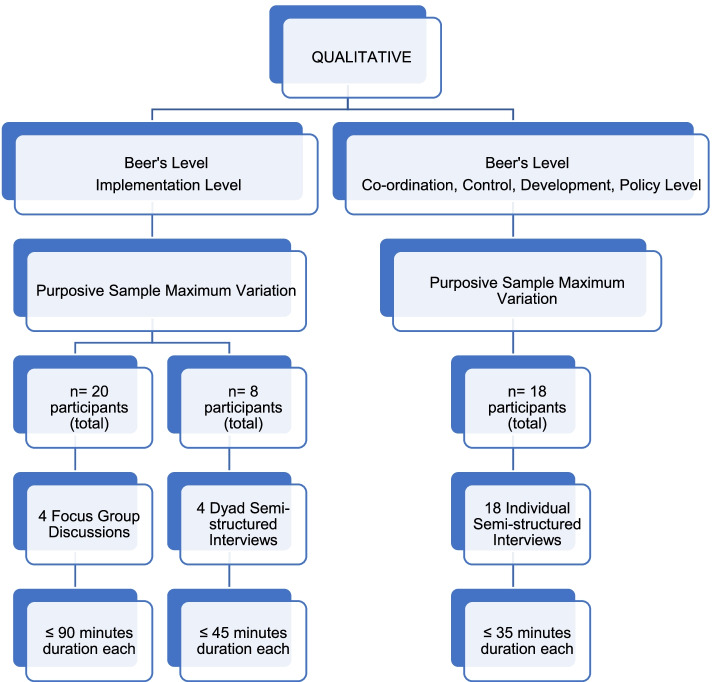
A diagram illustrating data generation and sampling

Data were generated concurrently through focus groups discussions and face-to-face individual/dyad sem-structured interviews. Four focus group discussions (*n* = 20) for not more than 90 min were conducted with participants at the implementation level. Owing to the availability and convenience for participants at the implementation level, four dyad face-to-face semi-structured interviews (*n* = 8) for not more than 45 min were conducted. Face-to-face individual semi-structured interviews (*n* = 18) were conducted for not more than 35 minwith participants at the coordination, control, development/intelligence and policy level function (based on Beer’s VSM) to avoid their influence on the focus groups where the majority of service providers were at the implementation functional level. Refer to Fig. 1: Diagram illustrating data generation and sampling.

### Data analysis

The audio recordings of the interviews and focus group discussions were transcribed verbatim to produce a written transcript by an independent transcriber prior to verification and editing by the first author for accuracy. Data were analyzed thematically [35] using a deductive approach. Codes were predetermined from the questions, aims and objectives of the study using Beer’s VSM [27] and resulted in four themes. NVivo Pro 12 qualitative data analysis software [36] guided the organization and further analysis of the data. The verbatim narratives of the participants were italicized and indented.

### Trustworthiness of the study

Four criteria were met to ensure the trustworthiness of findings, namely credibility, confirmability, dependability and transferability [26, 37]. This was achieved through the application of the following strategies, namely, to ensure a truthful representation and credible interpretation of the analysis, regular peer debriefing was carried out [26, 37–39]; identified themes were supported with extracts of verbatim narratives of the participants [40] which minimized the risk for bias; analyst triangulation was used [26] through two authors to identify possible shortcomings in the analysis process. Additionally, the study findings were examined against the existing literature [39]. A rich description of the context and methodological processes was exposed, with details for readers to determine the extent to which findings could be accepted and methods confirmed [38, 39].

### Ethical considerations

The study (Reference number BREC: BE274/17) was approved by the Biomedical Research Ethics Committee at the University of KwaZulu-Natal, South Africa. Due to the nature and sensitivity of information shared in this study, pseudonyms were used to maintain the confidentiality of service providers. Additionally, the participants’ designation, profession and place of work were not revealed (Table 1).

## Results

This study sought to explore service providers’ perspectives in aftercare service provision for PWSUD in a rural district. Service providers reflected on the state of aftercare services, observed barriers, acknowledged existing enablers and contributed to recommendations for aftercare service provision. Four themes emanated from the data sets, namely, (i) reflections of the interactional state of aftercare services and program content, (ii) identifying existing barriers to aftercare service provision, (iii) situating systemic enablers to aftercare service provision, and (iv) associated aftercare system recommendations.

### Theme 1: reflections on the interactional state of aftercare services and program content

#### Inadequacy of aftercare service provision

Service providers reflected on the inadequate state of aftercare in the district. In this rural district, the aftercare wasconsidered poor, executed within a fragile system, and if provided, lacked continuity and was deemed superficial at best, resulting in PWSUD being lost within the system.

*I can say that there is this missing point of follow up or continuity of care. (*Thandazile, Fieldwork and Implementation)

*There is still a lack of aftercare services to the people who have completed rehab, when they go out. Yes, they are still lacking aftercare so they find themselves coming back now and again in rehab because there is not much support for them … Yes, however, even the DSD is supposed to run an aftercare program … Ya they are supposed to because it is not happening but they have to (laughs). (*Carol, Management, Coordination and Control)

*They get discharged from rehab but they get lost we do not see them then they show up when relapsed*. (Jazzman, Fieldwork and Implementation)

*We can’t even find her. They are still hunting for her. (*Mngomezulu, Management, Control, Coordination and Implementation Control)

#### Notable effective and successful aftercare intervention

Service providers reported limited successful aftercare intervention i.e. among 45 service providers, only three aftercare success life stories reported.

Story 1

*But fortunately, I keep checking with the lady and she’s drawing closer. But I’m doing a lot of aftercare for the teenager, and fortunately the teenager has gone back to school this year and apparently, he’s doing well with the mother, though there are some elements of actually relapsing along the way. But with the care of the mother and also myself, we support. I also visit the school when I’m going to the clinic and stuff, just pass by the school just to check with the teachers. There’s a lot of improvement. (*Vika, Fieldwork and Implementation)

Story 2

*I think I visited the family because they knew that the patient had to stay for three months but I think he stayed for months. Then he had to explain at home why he came back earlier. So, I was the first one who was to talk to the family not to judge the patient because I knew that he had potential. He’s the one who came to my office and said no I’m tired of using drugs, so I want to stop now. Then we tried to apply for rehab at Newlands. Then when he came back the family was disappointed, but I had to talk with the family, no he’s okay provided we give him the support. Because he had potential, we were communicating now and again checking where he is, what he’s doing, yeah.* (Musa, Fieldwork and Implementation)

Story 3

*And I said to them no, it’s a collective work for us as therapists and also the institutions. The person, I mean even now I saw him, he’s functional and in the hospital where he is working. He is fully functional, I was actually doing some aftercare follow up with the supervisor in the ward that he is working and he’s doing exceptionally well. Even in the training, where he was supposed to go. They were taken to further their training in nursing and he was doing well. Even passing with flying colours, even as tutors in this college. So, you can really see that how substance abuse can rob us of our potential. (*Vika, Fieldwork and Implementation)

#### Validation of the types of aftercare services

There were inconsistencies in the provision of the limited aftercare services pertaining to home visits, family intervention, family reintegration services, school visits and individual counselling in this rural area.

*Preparation for the environment, home visit to the family to strengthen support system. Counselling for family and the affected member. Then a CCG (Community Care Givers) because we cannot always be there then if the CCG identifies the problem they report to us. (*Grace, Fieldwork and Implementation)

*We do individual counselling, they come here at the center. (*Joel, Fieldwork and Implementation)

### Recommended aftercare program content

#### The essentiality of family centeredness

Service providers recommended that aftercare be centered on the family, and the family should know their essential role in aftercare.

*As soon as they know that they have a more important role to play than the treatment center, they will then take part in a full way, in a fully pronounced way of the aftercare service. (*Mngomezulu, Management, Control, Coordination and Control)

*Because it’s also very important to strengthen family support because they stay with the family they don’t stay with us as health care workers. So, ours just ends here in the office, but I think we need to strengthen the family support. Even if we are not there. But the family will give support to the client. So that’s very important. (*Musa, Fieldwork and Implementation)

Family-centered aftercare should address broken relationships within the family.

*There are broken relationships because the family is affected, the society is affected, the family does not want this person back home. The society does not want this person back.* (Zinhle, Management and Control).

#### The pertinence of support groups in aftercare

Service providers reflected on the need for support groups during aftercare which were absent in their district. In addition, suggested that support groups should include family members because they also need support and a space to share their experiences.

*I also thought of support groups where they can talk about their experiences. This must include family because you find that families are in denial and some rely on traditional healers. So, if they come to support groups they can also learn as a family. (*Nickita, Fieldwork and Implementation)

#### Revisiting reintegration services in the system of care

Service providers maintained that reintegration services must endeavor to comprehensively reintegrate PWSUD within their context of family, workplace and community.

*Yes, of course aftercare is good because we can even report to employment centers where the person was employed, that the person has the right recommendation, that they must take him back. Another thing that makes (cause) failure is the companies, the workplace that employed this person, the negative attitude of taking this person back because he could have done a lot of bad things before he was sent to that place. They say “no we cannot take him back” in spite of the person being more knowledgeable than the people who are now replacing him. (*Mngomezulu, Management, Control, Coordination and Control)

However, reintegration is faced with a number of barriers, including stigmatization, therefore service providers should work with families and communities to facilitate reintegration.

*The referral social worker needs to work with the family as well as the society. This person has been through help so please give him a chance, a second chance person but we will be working with him in the recovery process because the recovery is not a year or two, it is a process*. (Zinhle, Management and Control)

#### Contextualizing the realities of vocational needs

Service providers admitted that most PWSUD have unmet vocational needs such as unemployment, job placement and skills development.

*Sometimes there are just basic issues, unemployment. Which are the things that you cannot do easily but at the local offices they have other programs like program 5. Then we said link them. (*Zinhle, Management and Control)

Service providers were of the view that the collaboration of departments in job placement could address unemployment needs.

*Departments should be able to talk and say so and so has been discharged, can we find a placement?* (Vika, Fieldwork and Implementation)

Additionally, addressing vocational needs should include skills development.

*I think maybe it’s not only counselling that they need; they also need some skills, give them skills because they use drugs most of them and because they are staying at home, they are doing nothing. (*Musa, Fieldwork, Implementation)

*In the aftercare program, if we can involve skills that we can do with them. That will create job opportunities for them. (*Carol, Management, Coordination and Control)

#### Sustaining relapse prevention

Relapse prevention was recommended through engaging in recreational activities, skills development and regular and consistent monitoring.

*It becomes easy for them to relapse if there is nothing that they are doing that is keeping them busy. Even playing soccer or some activities, being involved in other activities, it takes their minds away from drugs. (*Carol, Management, Coordination and Control)

*I think maybe if we do have a center, maybe a recreation center where they will come maybe once a week to have a support group there with them. (*Thando, Fieldwork, Implementation and Coordination)

Service providers also recommended aftercare that is chronic-orientated, which offers continual support or lifelong support for PWSUD.

*Another thing aftercare should not have a specific duration we must not let go of our clients but live with them and support them until they die* (Joel, Fieldwork and Implementation)

*They will always need intervention, regular follow-ups to prevent relapse.* (Vela, Fieldwork and Implementation)

### Themes 2–4: identifying existing barriers and situating existing enablers to service provision and associated aftercare system recommendations (Tables 2 and 3)

To deliver the aforementioned aftercare content, a comprehensive understanding of all components of aftercare service provision should be assessed, bearing the stakeholders in mind. In this study, different levels of service provision, as per the Beer’s VSM, namely implementation, coordination, control, intelligence/development and policy level, were explored [27]. Therefore, it is essential to classify these barriers and enablers at different levels to understand the contextual implications comprehensively.

**Table 2:** Theme 1 with Subthemes and Categories

Theme 1: Reflections on the interactional state of aftercare services and program content	
Subthemes	Categories
Inadequacy of aftercare service provision	• Superficial aftercare services • PWSUD lost within the system
Notable effective and successful aftercare intervention	• Success story 1 • Success story 2 • Success story 3
Validation of the types of aftercare services	• Home visits • The essentiality of family centeredness • Family reintegration services • School visits • Individual counselling
Recommended aftercare program content	• Affirming family centeredness • The pertinence of support groups in aftercare • Reintegration services of PWSUD • Aftercare to address vocational needs • Relapse prevention • Chronic orientated aftercare

**Table 3:** Themes 2, 3, & 4 according to VSM levels

Beer’s VSM Levels	Theme 2 Identifying existing barriers to aftercare service provision	Theme 3 Situating systemic enablers to aftercare service provision	Theme 4 Associated aftercare systems recommendations
Implementation level	• Internal Motivation of PWSUD • Family denial • Family’s limited knowledge of recovery process • Stigmatization of PWSUD • Poor community participation/partnerships in rehabilitation • Long waiting lists in ITCs • Unavailability of medication for withdrawal • Lack of education and training about SUD for service providers • Limited transport for service providers • Poor inter-sectoral collaboration • Lack of funding for aftercare services	• Team approach at hospitals and clinic level by DoH • High level of motivation of a PWSUD • Strong family support • Telephonic follow-ups from ITCs	Case manager or coordinator is required to coordinate aftercare services Teamwork in proving aftercare services • Teamwork should be facilitated through clinic card and CCG • Community partnerships facilitated through education cognizant of the cultural context.
Coordination level	Poor communication among stakeholders rendering services within the same community Limited awareness of each stakeholder’s roles, responsibilities and scope of practice. Poor communication between ITC & referring service providers	The necessity of collaborating with community caregivers.	Encouraging inter-sectoral collaboration among various sectors Inter-sectoral collaboration should be facilitated through war-room
Control level	Evaluation of SUD Services: poorly managed and monitored	Maximizing on war-rooms Considering a Ward-based approach	Strengthen monitoring and evaluative mechanism for aftercare services.
Intelligence/ development level	Limited accountability and reporting of NGOs to local institutions Absence of aftercare statistics in Provincial reports	Negligible support for SUD programs	Accountability of NGOs should also be at institutional level i.e. DSD facilities or hospital. Encouraging comprehensive details of SUD in reports
Policy level	SUD programs not prioritized by DoH and DSD NGOs reporting renewal at policy level only Lack of standard of care Lack of policy awareness	Policies in place	Revisiting the accountability of NGOs

### Implementation level

Service providers noted that some PWSUD lacked internal motivation towards recovery. Although service providers did not mention a highly motivated PWSUD as an enabler, the success stories for aftercare indicated that highly motivated PWSUD enabled service provision.

*He’s the one who came to my office and said no I’m tired of using drugs, so I want to stop now.* (Musa, Fieldwork and Implementation)

There were several barriers to the provision of aftercare to the family, namely family stigmatization, family denial and lack of knowledge regarding the recovery process.

*The family understands that this is a long-term problem, it’s not, we don’t fix it at the hospital. You’re admitted, you’re here and it’s expected that they will be fine, why are they not getting better? … Yes. You know there’s a, I find the families always come back, ‘why are they not getting better?’ ‘Why are they not stopping?’ And trying to make them understand the lifestyle change and it’s for the rest of their life. They may have to deal with this and the dynamics of the family that has to change.* (Dr K, Fieldwork, Implementation and Coordination)

Service providers identified barriers at the community level, such as stigmatization of PWSUD and poor community participation/partnerships in rehabilitation.

*I feel like not many people meet, so when you see a patient, they might come with psychosis, but they’ve only met their previous abuser so the stigma about being a substance abuser is still quite strong here, they are not open to talk about it. (*Zakithi, Fieldwork and Implementation*)*

Community partnerships were expressed as necessary in facilitating education that is culturally and contextually specific.

*I believe there can be educational workshops … You’ve got to equip them with knowledge and skills and how to deal with … but it must be culturally based, whatever information you are giving it must be sensitive to culture, cultural norms and values of that group because each group has got different norms. It doesn’t mean because you’re a Zulu, your norms are the same … Clan praises! (Laughs). (*Mngomezulu, Management, Control, Coordination and Control)

Service providers observed the lack of resources as a barrier in their respective sectors. These include staff shortages, a lack of ITCs in their district, lengthy waiting lists in ITCs situated in cities, unavailable medication for withdrawal symptoms, limited transport for home visits, and a lack of funding for aftercare services for NGOs.

*There is no funding allocated to aftercare, so even if you want to do aftercare, there is no budget. (*Palesa, Fieldwork and Implementation*)*

*From my department I felt we lack the constructive use of their time, and it will be very good to have groups. But due to a limited number of us, we don't have the human resources to carry through* (Zakithi, Fieldwork and Implementation)

Service providers also identified an urgent need for the training of social workers at the district level on SUD service provision in a South African context.

*One of the challenges we have is that the support for continuity of care as well as training, education, regarding how we proceed with continuity of care particularly with substance abuse, we do find that the Department of Health doesn’t really focus much on that. (*Dr K, Fieldwork, Implementation and Coordination)

Although service providers did not overtly express an interdisciplinary team approach as an enabler for service provision, service providers at some DoH institutions reported more comprehensive services at hospitals and clinics, using mental healthcare (MHC) teams. MHC teams enabled more robust communication and collaboration among disciplines within the same institution. The opinion of service providers is that poor communication among sectors could be minimized through promoting teamwork that can be facilitated through clinic cards and involvement of community caregivers (CCG).

CCGs are community-based and well acquainted with service users from the same community.

*We have mental health teams so we are integrated into the hospital. (*Nickita, Fieldwork and Implementation)

*Now it is better, we work with SORD (NGO), they are funded by DoH to provide mental health services. They do much treatment but focus on empowerment; they do follow up and support groups … We write referral letters to them. (*Grace, Fieldwork and Implementation)

*We also do lots of follow ups on the CCG’s since they are the ones who actually do a lot of baseline visits to the families to check as to if there is any improvement. Though sometimes you’ll find that the family will not give you all the information, but the CCG’s you’ll find they’ll give you all the information as to what is happening, is there any improvement, any changes that are there. (*Vika, Fieldwork and Implementation).

*Using the Clinic Card as a tool to communicate. Because everyone writes on this card even the CCG. Then we can all see what is written. Because when we do home visits we sometime go with the SW, OT and Psych nurse. I don’t know how realistic is that because we do not know.* (Thobile, Fieldwork and Implementation)

#### Coordination level

Service providers expressed that there is poor coordination of services characterized by poor communication and lack of monitoring.

*I am saying when they release a person from rehab sometimes, they do not tell us and that is a problem... We will only see a person when he is brought back when he has relapsed; this person, when was he released? That is another big problem... Uh … the communication between the rehab center and the treatment center and the treatment organization and the NGOs is not well monitored. (*Mngomezulu, Management, Control, Coordination and Control)

There is also poor coordination within the same sector, which indicates poor cooperation and interaction of sub-systems. There is miscommunication between ITCs (sub-system) and service providers at community level (sub-system).

*Even when they go to rehab they go there but when discharged, all the stakeholders I was telling are not aware. They go back home with no support. (*Thobile, Fieldwork, Implementation and Coordination)

*We still do not get any feedback unless a social worker from here is still liaising, client checking, that are you still okay; or maybe sometimes the client phones and “how are you doing”? “I am doing 1, 2, 3, 4,5 - giving the social workers feedback. (*Carol, Management, Coordination and Control)

To achieve a well-coordinated system of care, service providers were of the view that a case manager is required to assist with coordinating the service and facilitate inter-sectoral collaboration. The service providers revealed that existing collaborative structures such as school health teams and local drug action committees enabled the collaboration of stakeholders in prevention programs, but not in any treatment interventions.

*We work together with the school health team as well as with the local drug committee where we go to schools to do health education like awareness. (*Thandazile, Fieldwork and Implementation)

### Control level

Service providers acknowledged the inadequate monitoring of SUD services and the absence of statistics submission at the district level.

*No M and E for aftercare, even SUD we report on how many admitted. (*Nonhle, Coordination and Control)

*We do it on a small scale. It is touch and go … we group them in mental health stats, just reporting how many were SUD and how many were schizophrenic. (*Thobile, Fieldwork, Implementation and Coordination)

*No, we do not take any statistics of aftercare. Actually, nothing is reported about aftercare. (*Sydney, Fieldwork and Implementation)

*There should a very strong monitoring and an evaluation of SUD programs. More details for aftercare in the reports.* (Bhekani, Management, Coordination, Control and Development)

The existing program of war rooms (a meeting of multiple stakeholders working in a ward/a a particular community) appears to be an enabling mechanism for collaboration in prevention strategies.

*We use war rooms to communicate with other stakeholders where we identify cases together. (*Nonhle, Management, Coordination and Control)

*I believe we must also be using the war room groups. Yah. The war room groups because they are the ones that are going to report back to us*. (Mngomezulu, Management, Control, Coordination and Control)

In addition, DSD recently implemented a ward-based approach (each or several social workers are allocated to a ward to render all services) to service delivery which was said to be a facilitator for stakeholder collaboration.

*Ward-based approach is helping in a way although there are still challenges. (*Joanne, Coordination, Control and Intelligence)

### Intelligence level

Little support of SUD programs, characterized by inadequate provincial reporting tools, was noted as a barrier to service provision.

*There is support for other programs from provincial but very little for SUD services. Aftercare is not even recorded in monthly stats. (*Nonhle, Management, Coordination and Control)

*The reporting tool does not include aftercare at all. It does include the number of people sent to rehab though. SUD is not a priority … You find that we only report how many have been to rehab and it is not monitored. No statistics collecting it, so even when they have done it there is nowhere to report. (*Thembeka, Coordination and Control)

Service providers maintained that the accountability of state-funded NGOs should also be at local institutions.

*They run away from accountability, because these NGOs are funded by us … They are funded by Health, they are funded by DSD. (V*ika, Fieldwork and Implementation)

Additionally, service providers recommended that reports include SUD details such as aftercare and encompass the different/joint stakeholders instead of reporting in silos.

*Then by doing that, when you come to Province and report, it’s collective. (*Vika, Fieldwork and Implementation)

### Policy level

There was a noticeable lack of policy awareness on the service delivery (implementation) level, compared to service providers at the management level. As a result, the implementation service providers were unaware of specific policies guiding aftercare.

*No, we don’t have a specific standard. I can’t remember how I discharge them from or they discharge themselves, really. But I think once a month. (*Musa, Fieldwork and Implementation)

*No aftercare program we follow, no, no, there is no specific program. (*Jazzman, Fieldwork and Implementation)

In addition, service providers expressed that the DoH and DSD do not prioritize SUD programs. Instead, service providers are under pressure to meet targets for other competing programs.

*We have a lot of competing priorities. You see it has targets that are big very big, so social workers are chasing after them because they will report on them. So psychosocial services take a back seat because they will not report on them. (*Joanne, Coordination, Control and Intelligence*)*

Service providers at DSD expressed their frustrations about meeting targets of other programs, whilst for SUD, they were required to meet targets of prevention programs in the form of the number of people reached through awareness campaigns. In addition, the extensive reporting was time-consuming as opposed to rendering comprehensive services to their different client population. Notably, the targets they had to meet interfered with their duties.

*Even the community knows us that we no longer work but we are pushing targets. It is hard because if you do not meet the target you have to explain why. So, SUD takes a backseat*. (Joel, Fieldwork and Implementation)

## Discussion

### Reflections on the interactional state of aftercare services

The inadequate state of aftercare in the district is consistent with South African literature [1, 2, 13, 18, 22]; aftercare services are limited and, where available, were insufficient. In this study, the inconsistencies in the provision of the limited aftercare services affirm the previous studies, treatment services in SA are limited and mainly non-existent for the rural population [2]. Similarly, in an earlier study, only one out of five PWSUD had aftercare services post-discharge from an ITC in the same district [24]. Likewise, among 45 service providers, only three aftercare success life stories were reported in this study. However, these narratives demonstrated efficient teamwork at hospitals within the existing mental healthcare teams, which aided in the collaboration of various disciplines.

### Recommended aftercare program content

#### The essentiality of family centeredness

Service providers reiterated that aftercare should be centered on the family, a recurrent theme within available literature [17, 18, 41–43] and South African SUD policies [3, 23, 44, 45]. Social support from the family has been associated with positive outcomes in relapse [46, 47] and can assist with coping strategies for stress and dealing with triggers [47, 48]. Whilst the family is vital in aftercare, policy studies [3, 49] revealed that SA policies are ambiguous regarding family interventions. Nonetheless, service providers have expanded on family-centered aftercare intervention, which affords mending broken relationships for the reintegration of PWSUD and strengthening family support, creating a conducive environment for recovery and contributes to relapse prevention. This is consistent with findings from South African studies [17, 50] and international studies [51, 52]. Findings in this study suggest that to achieve a conducive environment, family education on SUD and the recovery process, especially building an understanding of the ‘chronic partners’ of SUD, is essential. Service providers indicated that family intervention should attend to family relationships, consistent with Groenewald & Bhana [49], who confirms that family relations are strained by the stress related to the user’s substance abuse. Therefore, family-centered aftercare allows a conducive space to address existing dysfunctional issues, which a social worker could facilitate. The familial patterns of dysfunctionality that may hinder recovery can be adequately examined in such a professional space; however, this could be a complex process [53].

#### The pertinence of support groups in aftercare

Service providers are aware of a critical absence of support groups in their district, although support groups are needed for PWSUD. Evidence from the literature indicates that involvement in support groups such as self-help groups contributes positively to reducing relapse and is an essential component of aftercare and treatment [23, 25, 46, 54]. Previous studies [54, 55], suggest that PWSUD who attended support groups had improved treatment outcomes for a longer time compared to those who did not. Laudet, Savage & Daneyal [46] study identifies community support services and association with the 12-step program as critical in maintaining sobriety for almost 12 years [46]. Furthermore, support groups offer several benefits, such as community reintegration [5], source of information and emotional support through peer support [56]. The peer support nature of the 12-step program provides information on health, employment, citizen restoration, a space for learning new skills and a conducive environment for establishing positive social relationships with others in drug and alcohol-free recovery environments [56].

In this study, service providers suggested that support groups include family members because they also need support and intervention. Generally, family members of a PWSUD mostly require to be equipped with coping strategies and knowledge on how best to be supportive [43], views which are also reinforced in support groups. In the previous study by Mpanza et al., [24], it was evident that family commitment to continued support group participation was a prerequisite for accepting a PWSUD [24]. Therefore, support groups such as mutual aid/self-help groups should be established in rural districts to benefit the family and PWSUD. Such groups can include both family and PWSUD and, in some sessions, be separated.

#### Revisiting reintegration services in the system of care

The Substance Abuse Act of 2008 emphasizes the importance of successful reintegration into home, family, workforce and community as an ultimate goal of aftercare intervention [25]. Similarly, service providers reiterated that aftercare program must endeavor to comprehensively reintegrate PWSUD within their context of family, workplace and community despite barriers such as stigmatization. Evidence from the literature indicates that reintegration is mainly hindered by stigmatization, a common phenomenon in SA [24]. However, community support services (CSS) play a vital role in reintegrating PWSUD into their community post-inpatient treatment [3, 54, 57] and may assist in mitigating barriers to successful reintegration.

#### Contextualizing the realities of vocational needs in aftercare

The opinion of service providers that PWSUD have unmet vocational needs to be addressed in aftercare is supported by SA literature [17, 18, 24, 42]. The various systems interact to produce an environment that is characterized by unemployment which has been linked to high relapse rates [42]. However, one must remain cognizant of the country’s 32.5% unemployment rate [58] that inevitably impacts vocational needs. To address this need, service providers suggested that PWSUD should be linked to job placement programs such as Program Five of the DSD to facilitate job placement. Program Five is a community development program focusing on sustainable livelihoods and job placement for the indigent and vulnerable population [59]. In addition, the collaboration of stakeholders is required to achieve job placement. Studies demonstrate the need for a collaborative approach in assisting PWSUD in finding employment [4], which aligns with the South African overall policy on SUD [12]. Specifically, departments such as Economic Development and Municipalities should collaboratively consider addressing the vocational needs of PWSUD. This is supported by the prescripts of the NDMP (2019–2024), which reiterates that stakeholders should cooperate in rendering services to PWSUD, including skills, personal and economic development, and creating employment opportunities and support for Small Medium Micro Enterprises (SMMEs) [12].

#### Sustaining relapse prevention

Service providers recommended aftercare programs to include relapse prevention. Evidence from the literature [17, 18, 50, 60–62] supports aftercare to include relapse prevention strategies to achieve sustained recovery. However, relapse is inevitable for some PWSUD, given the chronic patterns of SUD [63], noting that in SA, the majority of PWSUD relapse post-inpatient treatment care [1, 23, 34, 54]. Therefore, interventions should focus on relapse prevention and prepare for relapse so that its impact is minimized and adequately managed and accepted as part of the recovery process. Notably, the NDMP 2019–2024, UNODC, and WHO 2020 acknowledge SUD as a chronic, relapsing disease but does not provide clear guidance on how to manage relapse and lapse, which form part of a chronic disease pattern; instead, they only focus on relapse prevention, and not managing relapse [7, 12]. Thus, an aftercare program that is chronic-orientated, which offers continual support or lifelong support for PWSUD, is essential.

### Identifying existing barriers and situating existing enablers to service provision and associated aftercare system recommendations

Generally, in a rural context, various barriers exist and have been acknowledged in previous studies [2, 13]. Similarly, in this study, different levels of service provision, as per the Beer’s VSM, namely implementation, coordination, control, intelligence/development and policy level, were explored [27], which gave a comprehensive understanding of barriers and enablers with contextual implications.

#### Implementation level

A lack of internal motivation was noted as one of the barriers towards service provision and recovery, consistent with a previous study [64]. Internal motivation and readiness to change is integral to the recovery process [64]. Although a lack of internal motivation is considered a barrier to service provision, this should be noted with understanding that lack of motivation is part of the disorder that which the aftercare system should consider throughout the intervention. Therefore aftercare intervention should endeavour to continue motivate the PWSUD so that a lack of motivation is not a barrier/limitation to effective intervention.

Consistent with previous research [24, 43, 49, 53], SA policies [23, 44] and legislature [25], the study identified the family as an integral part of aftercare intervention. However, providing aftercare to the family is embedded with several barriers, namely family stigmatization, family denial and lack of knowledge regarding the recovery process. Such barriers affirm the notion that families of PWSUD require therapeutic intervention [65], which should address their specific needs and challenges. Moreover, to some families, substance use is a family legacy, with patterns that require examination within a therapeutic space for sustained change [65]. It was noted from the success stories that authentic and strong family support was integral in facilitating aftercare services. In this district, an earlier study by Mpanza et al., [24] also affirmed the necessity of strong family support for PWSUD.

At a community level, barriers such as stigmatization of PWSUD and poor community participation/partnerships in rehabilitation negatively impact service provision. Community stigmatization of PWSUD is common [18, 24, 66, 67] and typically influences community partnerships in rehabilitation. Thus, culturally appropriate community education would assist in eliciting community partnerships for the treatment of PWSUD [65] and should include efforts to minimize stigmatization [7].

Limited resources challenge service providers in rural areas within a largely non-conducive environment for recovery [65]. In this study, service providers reported a lack of resources as a barrier across all sectors. The majority of services are offered by the DSD, [2] thus, a needs assessment may provide direction as to how the mobilization of resources could be implemented. As noted in the literature [2], funding is generally an issue, particularly for NGOs; for instance, in this study, NGO service providers complained about the lack of funding and budget allocations, even though they were expected to render comprehensive services inclusive of home visits. Another concomitant aspect is essential staff shortages which hamper comprehensive care. In addition, ongoing staff development and training on SUD is critical. Consequently, a study by Burnhams et al. [2] also identifies an urgent need for the training of social workers at the district level on SUD service provision in a South African context. Similarly, the international standards for the treatment of SUD by UNODC and WHO also recognize the need for the training of service providers in SUD management [7].

#### Coordination level

Service providers’ perspectives revealed that SUD treatment services are inadequately coordinated within and among respective sectors. Poor communication lends itself to inefficient coordination. Despite rendering services to the same community and engaging the same person, they remain unaware of each other’s role. In SA, working in compartmentalized sectors is common within the treatment system of SUD [16, 68], despite Acts [25] and policies [7, 23, 44] promoting inter-sectoral collaboration. In some cases, poor coordination is within the same sector, despite policy guidelines available [44]. The cooperation and interaction of sub-systems of the overall treatment system influence the commitment to aftercare for PWSUD [69]. In addition, poor coordination between ITCs and service providers at the community level contributes to increased relapse rates [13]. Therefore, a coordinated, comprehensive system of care that contributes towards the prevention of relapse and complications aligned to treatment goals by UNDOC and WHO should be the desired goal [7]. In this study, a case manager was proposed as a solution to assist in achieving a well-coordinated system of care and facilitate inter-sectoral collaboration, which is in line with the national minimum norms and standards [44]. However, coordination of service remains an ongoing challenge.

#### Control level

The study identified the inadequate monitoring of SUD services at the district level as a barrier to service provision. Whilst aftercare is a sub-system of the SUD treatment system; it is also a sub-system in itself. A recent study by Mpanza et al. [3] revealed that SUD services’ inadequate monitoring and evaluation may be linked to vague policy directives. In addition, no statistics nor reports for aftercare services are submitted at the provincial level. Inevitably, this lack of record keeping will negatively impact the aftercare system.

Although enablers were not easily identified in this study, the existing program such as war rooms and DSD ward-based approach to service delivery appeared to be an enabling mechanism for collaboration in prevention. Enhancing such programs to extend to treatment and aftercare could maximise available resources for rural communities [65].

#### Intelligence level

The study identified little support of SUD programs at a provincial level, characterized by inadequate provincial reporting tools, as a barrier to service provision. Service providers recommended that reports include SUD details such as aftercare and encompass the different/joint stakeholders instead of reporting in silos. This could foster collaborations and inter-sectoral service delivery as expressed in the NDMP of 2019–2024 [12], Substance Use Act [25], and the UNODC and WHO International Standards for the Treatment of Drug Use Disorders [7]. In addition, service providers proposed that reporting and monitoring of NGOs rendering services at the community level should be extended to the local institutions such as hospitals and service centres as oppose to be monitored at the provincial level only. This skewed accountability hinders progress at an intelligence level.

#### Policy level

In this study, there was a lack of awareness of specific policies guiding aftercare at the implementation level. The lacunae in such policy awareness influence service delivery as services are aligned with policy directives. Policies such as the National Minimum Norms and Standards for Inpatient Treatment [44] should be familiar to service providers. In addition, SUD programs are not prioritized by the DoH and DSD, and the extensive reporting is time-consuming. Both targets and comprehensive reporting interfered with service providers duties. The heavy caseloads and excessive paperwork of service providers have reportedly interfered with their clients’ quality of service [65], to the extent of neglect of other services, prioritizing other programs seem common in the public sector. Mpanza and Govender [1] also affirm that mental health and SUD programs were not prioritized in this district due to priority programs such as HIV and TB. Whilst prioritizing certain programs is understandable, for instance, this particular district has a high prevalence of TB and HIV, a balanced and equitable distribution of resources to all programs as per policy directives should be a guide. In addition, evidence-based practice should be promoted in service planning as indicated by UNODC and WHO 2020 [7].

### Study limitations

The study explored the perspectives of service providers in aftercare service provision. The study was conducted in one outlying rural district away from cities where ITCs are located. Hence district/s closer to ITCs may be of the same rural context but vary in experience due to their proximity to towns compared to this district. Future studies should consider exploring more than one district to expand the comprehensive understanding of aftercare service provision in many districts.

## Conclusions

The intersecting systemic complexities of providing aftercare services in a rural context in SA was evident in this study, irrefutably demonstrating the weaknesses and inadequacies within a fragile system. Aftercare services were at best superficial, such that PWSUD were lost within the system of aftercare. Where it was present, service provision followed an acute model instead of lifelong intervention as prescribed by overall policy. However, the limited success stories and effective teamwork at hospitals within the mental healthcare teams demonstrated the value and strength of integrating SUD services with the mental healthcare system. Minimal enablers exist for service provisions in this rural district, such as integrated SUD services to the mental healthcare system at the implementation level and existing multiple stakeholder collaborative programs such as war rooms and Operation *Sukuma Sakhe*^1^ at the control and coordination level. These appeared to stimulate the collaboration of multiple stakeholders to a limited extent, therefore, it should be strengthened and extended to SUD treatment services at all levels of service provision. Service providers were continuously faced with numerous systemic barriers at all levels of service provision. Additional barriers were identified at the implementation and policy level. A key barrier was the low prioritization of SUD in government departments, where competing priority programs took precedence over SUD services. As a result, the various shortfalls of the SUD system were characterized by inadequacies in the aftercare system. To strengthen the aftercare system, policies with enforcement are required for aftercare services and outcome-based monitoring and evaluation linked with specific indicators. Moreover, a model of aftercare that is family-centered and sensitive to the rural context, encompassing both relapse management and prevention, is responsive to individual needs and evidence-based, integrated into the existing systems of service provision and one that encourages stakeholder collaborations, could also strengthen and sustain the aftercare system and service provision. To achieve a functional aftercare system, the perspectives, experiential knowledge and insight of service providers and PWSUD with their families as essential stakeholders should be considered for further policy developments, service delivery strategies and effective aftercare model development.

## Data Availability

The data generated during the current study are not publicly available to protectof the confidentiality for participants. Data is available upon reasonable request from the first and corresponding author.

## Abbreviations

CCG: Community Care Givers
DoH: Department of Health
DSD: Department of Social Development
ITCs: In-patient Treatment Centers
MHC: Mental Healthcare
NGO: Non-Governmental Organization
nGAP: New Generation of Academics Programme
PWSUD: Persons with Substance Use Disorder
KZN: KwaZulu-Natal
SA: South Africa
SUD: Substance Use Disorder
SMMEs: Small Medium Micro Enterprises
UNODC: United Nations Office on Drugs and Crime
VSM: Beer’s Viable Systems Model
WHO: World Health Organization

## Footnotes

1 Operation Sukuma Sakhe (Stand up and build) refers to the integrated service delivery model bringing together all service delivery stakeholders to provide services in an integrated manner.
